# Effectiveness of school-based CPR training among adolescents to enhance knowledge and skills in CPR: A systematic review

**DOI:** 10.4102/curationis.v45i1.2325

**Published:** 2022-11-18

**Authors:** Nombulelo E. Zenani, Bashir Bello, Matsipane Molekodi, Ushotanefe Useh

**Affiliations:** 1 Department of Nursing Sciences, Faculty of Health Science, North-West University, Mafikeng, South Africa; 2 Lifestyle Disease Research Entity, Faculty of Health Science, North-West University, Mafikeng, South Africa

**Keywords:** adolescents, cardiopulmonary resuscitation, knowledge, skills, training

## Abstract

**Background:**

Cardiac arrest is responsible for 5% – 10% of all deaths among children age 5–19 years; therefore, strategies to prevent poor outcomes post cardiac arrest among children are critical within schools.

**Objectives:**

The purpose of this study was to systematically review the effectiveness of cardiopulmonary resuscitation (CPR) training on CPR knowledge and skills among adolescent school children.

**Method:**

This systematic review was conducted and reported using the Preferred Reporting Items for Systematic Reviews and Meta-Analyses (PRISMA) guideline. The complete bibliographic databases of PubMed, Cochrane Library, CINAHL and Web of Science were searched from January 2012 to August 2021. Included studies met all the eligibility criteria. The Effective Public Health Practice Project Quality Assessment Tool for Quantitative Studies (EPHPP) and Mixed Method Appraisal tool were used to appraise the quality of the included studies.

**Results:**

Fourteen studies were included in the review, and 5418 participants were found in the databases. The studies were mainly conducted during the last decade, which suggests that the public’s attention has been directed toward training schoolchildren in CPR. The most common interventions were taught in video simulation training courses. They also used subjective assessments to evaluate the participants’ knowledge and skills.

**Conclusion:**

Findings from this systematic review reveal that CPR training within school settings effectively promoted a change in CPR knowledge and skills among adolescents. Hence, continuous training of CPR among schoolchildren should be encouraged by policymakers, school authorities, parents and teachers to optimise the prompt usage of the skills in any cardiac event. However, a high-quality randomised controlled trial would enhance the strength of evidence in this area.

## Introduction

Out-of-hospital cardiac arrest (OHCA) has remained a significant public health concern. Out-of-hospital cardiac arrest accounts for a substantial number of death worldwide (Pivač, Gradišek & Skela-Savič 2020). Every year, over 700 000 people in Europe and the United States of America suffer from OHCA. The survival rate remains alarmingly low, ranging from 5% to 10% (Wingen et al. 2018). In Australia, an estimated 24 373 cases of OHCA were reported in 2017, with a survival rate of 12% (Pivač et al. 2020). In London, from May 2012 to December 2017, 1055 patients were reported to have OHCA (Reveruzzi, Buckley & Sheehan 2016). These studies prove that OHCA remains the leading healthcare concern worldwide, influencing the morbidity and mortality rates of the population across the world. About 90% of patients who are victims of OHCA die before reaching the hospital; this can be attributed to poor knowledge of cardiopulmonary resuscitation (CPR) (Van Rensburg et al. 2021). According to the Utstein definition, cardiac arrest is a sudden cessation of cardiac mechanical function as evidenced by the absence of detectable pulse, absent or gasping breath and loss of consciousness (Sandroni et al. 2007).

The CPR intervention is essential in improving the chances of survival of cardiac arrest patients in and out of the hospital setting. Early initiation and good quality of CPR by bystanders and automated external defibrillator use are crucial for saving patients in cardiac arrest. However, the implementation rate for bystander CPR is reported to be low (Xu, Zhang & Chen 2017). Furthermore, resuscitation limits neurological damage caused by cardiac arrest when promptly performed (Chocron et al. 2021). The key strategies to improve survival from OHCA are described as the links in a chain of survival; early recognition of OHCA requires observing signs of poor circulation such as pale face and cold extremities, no breathing and no pulse. The second stage is activating the emergency response by calling the emergency response team for advanced medical assistance, followed by early CPR with chest compressions. Cardiopulmonary resuscitation aims to provide blood circulation to the brain, heart and other vital organs deprived of the circulation caused by cardiac arrest. The last action is rapid defibrillation to rectify the abnormal heart rhythm caused by the cardiac arrest and post-CPR care in the hospital (Chocron et al. 2021). Collectively, these interventions provide the best opportunity to improve OHCA survival.

In OHCA, these critical linked interventions mostly rely on a layperson, who is often the first on the scene (Chocron et al. 2021). However, sporadically when the layperson witnesses the cardiac arrest and commences with the chain of survival links, there is a greater chance that the patient can regain signs of circulation when the emergency response team arrives (Stassen et al. 2021). Therefore, it would be immensely beneficial if these life-saving skills were introduced at places of employment and learning. Pharaoh, Frantz and Smith (2011) report that life skills are a group of psychosocial competencies and interpersonal skills that help adolescents make informed decisions and deal effectively with the demands and challenges of everyday life. Life skills, according to Hawkins et al. (1999), are a great way to empower adolescents to make educated and responsible decisions about their own lives and well-being, which may be directed toward personal actions. Therefore, introducing CPR training to adolescents who are in school has served as an effective intervention to improve CPR rates and increase survival rates of OHCA (Cave et al. 2011).

Furthermore, early CPR training enhances the safety culture of schools and the role of the teachers to the pupils. For example, should one of the adolescent learners experience cardiac arrest on the soccer field while the teacher is absent, the pupil trained in CPR can save another pupil’s life by acting promptly according to the chain of survival links. This can result in long-term structural changes of accountability and confidence in performing in such emergency situations. Furthermore, the pupils trained in CPR can serve as CPR multipliers, as they may pass on the acquired CPR knowledge and skill to family members and friends.

As recommended by the American Heart Association (AHA), the drive for CPR training is a golden opportunity to improve school and community health (Nordheim 2019). Thus, this systematic review aimed to systematically review the effectiveness of CPR training in schools and its impact on CPR knowledge and skills among adolescents.

## Study aim

The aim of this study was to systematically review the effectiveness of CPR training in schools and its impact on CPR knowledge and skills among adolescents.

## Methods

This systematic review was conducted and reported using the Preferred Reporting Items for Systematic Reviews and Meta-Analyses (PRISMA) guidelines (Moher et al. 2010). The protocol was registered in the International Prospective Register of Systematic Reviews (PROSPERO) with the reference number CRD42021266518. According to Aromataris and Pearson (2014), a systematic review aims to provide an unbiased synthesis of relevant studies in a single document. A systematic review consists of the inclusion of study selection, critical appraisal and data extraction, which must be conducted by independent reviewers to reduce the risk of error, which may affect the results of the review. The systematic review in the context of this study was regarded as the best choice to employ because it enables the authors to explicitly comprehend the depth of existing knowledge on CPR training, CPR knowledge, CPR skill retention among adolescents and the impact it has on adolescents in school-based settings (Aromataris & Pearson 2014).

### Search strategy

Independent reviewers B.B. and N.E.Z. searched the databases of PubMed, CINAHL and Cochrane Library to identify studies that were published from January 2012 until August 2021. Medical Subject Headings (MeSH) and free text terms were used in the search to the find studies. For the Web of Science search, a combination of terms and truncation was used. Only human studies that were peer reviewed and published in English were considered. A manual search was also conducted from the references of the studies and other sources to complete the review. See full search strategies in Figure 1.

**FIGURE 1:**
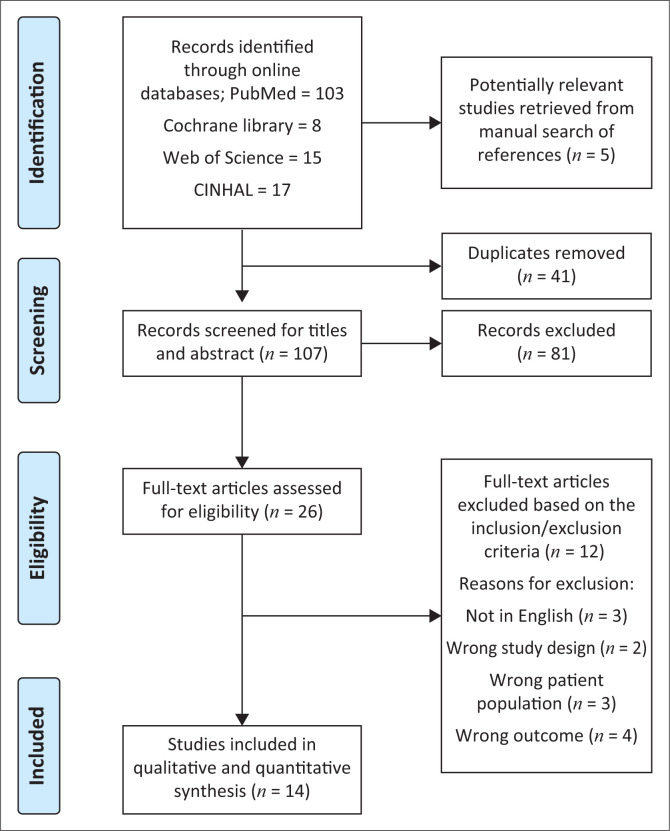
Characteristics of included studies.

### Study eligibility criteria

Studies were eligible for inclusion if they examined the effects of CPR programs or basic life support (BLS) training (specifically, CPR programs), on CPR knowledge retention, skills, attitudes and perceptions of CPR usage in adolescents between the ages of 12–18 years (Table 2). To be included in this review, studies had to meet the following eligibility criteria:

- **Population:** generally adolescent schoolchildren aged 12–18 years.
- **Intervention:** interventions or programs which include CPR or BLS.
- **Outcome:** objectively or subjectively measured knowledge of CPR, CPR skills, safety and prevention of cardiac arrest skills, attitude and adolescents’ perceptions of CPR usage.
- **Design:** randomised and nonrandomised trials were included; cross-sectional and qualitative studies, reviews, meta-analyses and guidelines were excluded.

### Study selection

All identified articles were exported into EndNote (Clarivate Plc, London, United Kingdom) to remove duplicates and then uploaded onto the Covidence online software (Veritas Health Innovation Ltd, Melbourne, Australia) for screening. The identified articles were initially screened based on titles and abstracts, based on two researchers’ eligibility criteria (B.B. and N.E.Z.). Then the same researchers screened the full-text copies of articles that scaled through the title and abstract screening after removing irrelevant articles. A third author (U.U.) resolved potential areas of disagreement on whether to include or exclude articles. Finally, the references of articles identified through database searches were examined to determine any further potentially relevant studies. See complete search strategies in Figure 1.

### Quality assessment of studies

The Cochrane Collaboration recommended tool for quantitative studies, Effective Public Health Practice Project Quality Assessment Tool for Quantitative Studies (EPHPP), was used to appraise the quality of the included quantitative studies (EPHPP 2009). In this study, two researchers (B.B. and N.E.Z.) reviewed each study using the EPHPP tool. The tool consists of eight items assessing selection bias, study design, confounders, blinding, data collection methods, withdrawals and dropouts, intervention integrity and analysis. Each item was rated as 1 = strong, 2 = moderate or 3 = weak. For any study that failed to present a clear and transparent description, the item was rated 3 = weak (EPHPP 2009). A ‘strong’ study connotes studies without weak ratings on any of the eight items. Studies with one weak rating were termed ‘moderate’, and two or more weak ratings connote ‘weak’ studies. In the included 14 studies, one used mixed methods. The researchers used the Mixed Method Appraisal Tool (MMAT) to appraise the study. The MMAT is designed to appraise systematic mixed studies reviews and also permits appraisal of the methodological quality of five research categories, namely qualitative, quantitative, randomised control trials (RCTs), nonrandomised, quantitative descriptive and mixed methods studies (Hong 2018). The results of the quality assessment are summarised in Table 1.

**TABLE 1:** Quality appraisal summary of the included studies.

Study	Selection bias	Study design	Confounders	Blinding	Data collection methods	Withdrawals and dropouts	Overall study quality
Barsom et al. (2020)	1	1	1	3	1	1	Moderate
Cortegiani et al. (2017)	1	1	1	3	1	1	Moderate
Fonseca Del Pozo et al. (2016)	2	1	1	3	1	1	Weak
Haseneder et al. (2019)	1	1	1	2	1	1	Moderate
Li et al. (2018)	1	1	1	3	1	1	Moderate
Meissner et al. (2012)	1	2	2	3	1	1	Weak
Nord et al. (2017)	1	1	1	3	1	1	Moderate
Mathew et al. (2020)	1	2	1	3	1	1	Weak
Onan et al. (2019)	2	2	1	3	1	1	Weak
Paglino et al. (2019)	1	1	1	3	1	1	Moderate
Suss-Havemann et al. (2020)	1	1	1	1	1	1	Strong
Tsai et al. (2019)	1	2	1	3	1	1	Weak
Wingen et al. (2018)	1	1	1	3	1	1	Weak
Pivač et al. (2020)	1	2	1	3	1	1	Moderate

### Ethical considerations

This article followed all ethical standards for research without direct contact with human or animal subjects.

## Results

A total of 143 articles were retrieved from the databases. After the 41 duplicates were removed, 102 were screened for abstract and title, and 81 were excluded. Twenty-one of these were considered for full-text eligibility, and 12 were rejected because they did not meet the eligibility criteria. Fourteen studies (nine RCTs and five non-RCTs) were included in this systematic review. The majority of the studies were of moderate to weak quality.

### Characteristics of the included studies

Table 1 demonstrates the characteristics of the included studies. The total number of participants in the 14 included studies was 5418. All of the studies were carried out within the last decade (January 2012 – August 2021), indicating the recent attention on the need to train schoolchildren on how to carry out CPR among their peers and others outside the school to save lives. Among the 14 included studies, 9 were RCTs (Barsom et al. 2020; Cortegiani et al. 2017; Fonseca Del Pozo et al. 2016; Haseneder et al. 2019; Li et al. 2018; Nord et al. 2017; Suss-Havemann et al. 2020; Wingen et al. 2018), while the remaining five were nonrandomised studies. In the 14 included studies, 11 of the studies were conducted in Europe, with only three from Asia (Li et al. 2018; Mathew et al. 2020; Tsai et al. 2019). There was no study carried out in Africa. According to Kraus et al. (2016), the sub-Saharan African countries have a high prevalance of cardiac arrest with poor clinical outcomes, which are associated with recurrent hospitalisation and a substantial healthcare expenditure. This is in contrast to Western countries, where cardiac arrest affects mostly older persons; in Africa, even adolescents are at risk. The limited literature on school-based CPR intervention in Africa is alarming. In the participating schools, the interventions used to assess the effects of CPR among adolescents in the majority of the studies were mainly instructor-led basic CPR training with video simulations of chest compressions or basic CPR theoretical training. Few studies used virtual reality and video gaming to teach CPR to students (Barsom et al. 2020; Fonseca Del Pozo et al. 2016). The outcome measure used by most of the studies was subjective questionnaires used to assess CPR knowledge and skill. For example, Cortegiani et al. (2017) used Laerdal QCPR 1 software (Laerdal, Stavanger, Norway) to measure chest compressions pre- and post-CPR training. All the included studies had a high risk of bias in the blinding of the participants and assessors. Table 2 details the evidence of synthesis and the characteristics of the included studies.

**TABLE 2:** Evidence of synthesis and characteristics of the included studies.

Authors (year) and country	Study design	Age range (years)	Number of participants	Intervention	Control	Outcomes measured	Findings	Conclusion
Barsom et al. (2020) Netherlands	RCT	16	40	Virtual reality (VR)	Standard training	A questionnaire concerning statementson useability, self-confidence, content and overall quality	The VR group had a significantly higher increase in correct answers in comparison with the standard group.	The use of VR training for CPR training appears to be an effective learning method for nonmedical students and may be of great value skilling high school students in becoming adequate CPR providers
Cortegiani et al. (2017) Italy	RCT	17–18	144	Real-time feedback during chest compressions with the guidance of an instructor	Based on standard instructor-based feedback	The primary outcome of vthe study was the compression score calculated by Laerdal QCPR1 software 7 days from the training	Students in the QCPR group had a significantly higher compression score compared to the SF group. Students in QCPR group performed significantly higher percentage of fully released chest compressions and rate	Training for chest compressions based on real-time feedback software guided by an instructor is superior to instructor-based feedback training for chest compression skills
Fonseca Del Pozo et al. (2016) United Kingdom	RCT	12–14	122	CPR song and video	Standard	To assess the acquisition of theoretical knowledge, a CPR questionnaire was used	No significant difference between experimental and intervention group in terms of CPR knowledge 1 month post intervention. However, at 8 months there were significant differences between the groups	The study showed that incorporating the song component in the CPR teaching increased its effectiveness and the ability to remember the CPR algorithm
Haseneder et al. (2019) Germany	RCT	10–17	460	BLS training conducted by emergency physicians	BLS training conducted by medical students	Subjective assessment of BLS knowledge and self-confidence	BLS knowledge increased from 5.86 to 9.24 and self-confidence increased from 8.70 to 11.29. After 9 months, knowledge retention was good but self-confidence significantly declined	BLS training led to short-term gains in knowledge and self-confidence. Although knowledge was retained at 9 months after the training session, self-confidence significantly decreased
Li et al. (2018) China	RCT	13–14	1093	CPR, instructor-led	Nil	The primary outcome f the investigation was the correct rate of CPR knowledge-related items	After training, bystander CPR theory was significantly improved, and students reached an 85% – 100% performance rate in a simulated BLS scenario	Schoolchildren in China have a poor pretraining knowledge of bystander CPR. However, with training, there was a significant improvement in the basic theory and skills of CPR
Meissner et al. (2012) Germany	Longitudinal prospective cohort study	14–15	132	CPR, instructor-led	Nil	BLS performance with a yes-or-no checklist in each practical assessment	Before the training, 29.5% of students performed chest compressions as compared to 99.2% post-training. At the 4-month follow-up, 99% of students still performed correct chest compressions. The overall BLS performance score was also statistically increased from 4 to 10	BLS training in high school seems highly effective considering the minimal amount of previous knowledge the students possessed
Nord et al. (2017) Sweden	RCT	12–13	587	CPR training with a practical test, including feedback (T), or CPR training with reflection and a practical test, including feedback	Basic CPR training only (O)	The primary endpoint was the total score for the modified Cardiff test at 6 months	At 6 months, the T and O groups scored 32 (3.9) and 30 (4.0) points, respectively (*P* < 0.001), while the RT group scored 32 (4.2) points (not significant when compared with T)	A practical test including feedback directly after training improved the students’ acquisition of practical CPR skills. Reflection did not increase further CPR skills
Mathew et al. (2020) India	Prospective interventional study	12–16	810	Basic CPR training and video demonstration	Nil	CPR knowledge assessment was done with a 10‑statement questionnaire covering 3 aspects: basic knowledge, theoretical aspects and algorithm of hands‑only CPR	68% of middle school students, 79% of secondary school children and 82% of senior secondary school children performed correctly chest compressions in terms of rate, depth and duration	Theoretical training of CPR can be started at the middle school level, and practical training can be incorporated in the school curricula from secondary school
Paglino et al. (2019) Italy	Interventional study	14–19	318	CPR video-based training	Nil	A theoretical questionnaire, made up of six questions on CPR and a practical test of 1 min of compression- only CPR on a Laerdal Resusci Anne Wireless Skill Reporter mannikin	Three months post intervention, 98% of the students knew when to perform CPR and 97% were competent in the BLS sequence. Post training after 6 months, 98% knew the theoretical skills of BLS, 96.5% knew when to perform, 95% were competent on the practical test with correct chest compression rate, depth and hand placement	Training schoolteachers to teach students in high schools using video-based training provides a good retention of both theoretical and practical skills after 3 and 6 months
Onan et al. (2019) Turkey	Quasi-experimental study	12–17	83	CPR, instructor-led	Standard self-training	BLS knowledge test using 15 multiple-choice questions and CPR performance	Instructor-led CPR increased the BLS knowledge significantly	Simplified BLS training increases BLS knowledge scores; thus, BLS training should be a mandatory component of the high school curriculum
Pivač et al. (2020) Slovenia	Mixed methods study	12.5–14.5	746	Basic CPR training	Standard	Knowledge of CPR was measured using a structured questionnaire consisting of four sections with 27 nominal-level binary questions	Significant progress in CPR knowledge was noted after training implementation, with increase in the following variables: attitude in helping others (*p* = 0.001) and self-confidence (*p* = 0.001)	Early CPR training is recommended in school as it will raise awareness of the responsibility to help others provide quality bystander CPR; thus, it should be mandatory in the school curricula
Suss-Havemann et al. (2020) Germany	RCT	12	307	Basic CPR training with a skill-testing scenario	Standard	Practical skills were evaluated during a 3-min scenario testing using a nine-point standardised checklist	This study could not measure a higher self-efficacy for helping in cardiac arrest of the students participating in the intervention compared to the control group (*p* = 0.135)	The study could not demonstrate that self-regulated learning supports higher self-efficacy for helping in cardiac arrest in students
Tsai et al. (2019) Taiwan	Quasi-experimental pre-post study	12–17	336	50-minute CPR and automated external defibrillator training	Standard	CPR knowledge was tested using a questionnaire with 10 items	Improved knowledge of emergency response, correct CPR at home and willingness to help	Early CPR training in schools significantly improves immediate knowledge of CPR in school children and empowers them to act
Wingen et al. (2018) Germany	RCT	14–18	424	90-minute basic CPR training	Standard	CPR knowledge was tested using a previous validated questionnaire	School children in the intervention group showed a significantly higher level of knowledge (*p* < 0.001) and self-confidence (*p* < 0.001) compared with controls.	CPR training improves the level of knowledge and self-confidence in schoolchildren

RCT, randomised controlled trial; CPR, cardiopulmonary resuscitation; QCPR, quality cardiopulmonary resuscitation; BLS, basic life support; SF, standard with feedback; RT, reflection with training; VR, virtual reality.

### Effects of interventions on cardiopulmonary resuscitation knowledge and skills

Only one study (Suss-Havemann et al. 2020) out of the 14 included studies reported a lack of significant effect on CPR knowledge and skill retention among adolescents post CPR training. This may be because the study measured self-efficacy in helping students with cardiac arrest and did not directly measure the post-training effect on knowledge of CPR. In resuscitation, self efficacy is defined as the judgement of perceived capability to organise and execute CPR (Roh et al. 2012). According Riggs, Franklin and Saylany (2019), self-efficacy may be associated with improved skills; however, that varies from one population to another. The same authors emphasised that skills detoriate more rapidly than knowledge, and frequent training to effectively ensure self-efficacy is advised, especially among adolescents within the schools (Riggs et al. 2019). In the study by Suss-Havemann et al. (2020), a nine-point standardised checklist was used to measure self-efficacy using practical skills evaluated during a 3-min scenario testing. This checklist does not explicitly measure the CPR knowledge or skills, but rather it assesses the students’ self-efficacy by measuring their readiness to help in three domains: (1) in general, (2) helping in cardiac arrest and (3) diminished emotional arousal to cardiac arrest; the assessment used a four-point Likert scale as follows: 1 = not at all true; 2 = hardly true; 3 = moderately true; 4 = exactly true.

## Discussion

This review showed that CPR training within school settings among adolescents effectively enhances adolescents’ knowledge, skills and confidence in providing CPR for peers and bystanders with OHCA. The only strong quality RCT in this systematic review demonstrated a lack of self-efficacy scores for helping students with OHCA. This could be because the authors measured self-efficacy in assisting students with cardiac arrest, not participants’ CPR knowledge post-training. Previous studies (Pivač et al. 2020; Reveruzzi et al. 2016) had shown that introducing early CPR training among adolescents is essential in enhancing their CPR knowledge, readiness to help others and confidence to provide CPR to peers and bystanders with OHCA.

Western countries have shown that children can learn these basic CPR skills and prevent sudden OHCA. However, findings from this review showed that no country in sub-Saharan Africa had carried out CPR intervention in school settings among adolescents.

Unfortunately, in sub-Saharan Africa, approximately 5 million deaths each year are attributable to conditions that could have been addressed by prehospital care, which is underdeveloped in most low- and middle-income countries, such as cardiac arrest (De Buck et al. 2020). The AHA recommends that CPR training be offered to children in schools starting at 12 years (Weidenauer et al. 2018). The present review indicates that most schools in Africa are yet to key into this rule.

The studies revealed that the training intervention typically targeted adolescents from ages 14 to 18, mainly due to their physical structure and maturity to demonstrate the skill required and retain the theoretical component of the training. In the 14 studies included in this review, the duration of activity ranged from 50 min to 4 h, including the practical and didactic components. Research by the AHA supports effective CPR training intervention; the longer the duration, the more significant improvement is observed in knowledge and retention of information (Cave et al. 2011).

In this review, the most common interventions used in adolescents’ CPR training were instructor-led basic CPR training with video simulations of chest compression, basic CPR theoretical training with mannikins and video gaming to teach CPR to students (virtual reality). In addition, the AHA introduced the Blended Learning Program in 2015. This concept involves the use of video- and computer-based modules to deliver self-paced, online training in CPR. Unfortunately, while it has been widely used in the United States, it is not yet commonly utilised in Africa because of the coronavirus disease 2019 (COVID-19) pandemic. This review is still important and relevant, because the authors recognise that adolescents will become elders in the future if they are taught and can perform CPR in an emergency such as cardiac arrest; they may be able to save the lives of cardiac arrest victims and educate their younger siblings, increasing bystander rates.

This systematic review revealed that most adolescents had limited knowledge and skills on CPR, as observed in the pretest questionnaires distributed in all studies where participants had no previous experience of CPR. Furthermore, out of the 14 included studies, the adolescents demonstrated no bystander effect before training, a psychological phenomenon involving an unwillingness to assist a person with cardiac arrest. The main reason was limited knowledge and fear of causing further injuries to the cardiac arrest victim.

The studies’ predominant tool for outcome measure was questionnaires combined with technical skills assessed by the instructors after the CPR training interventions (Haseneder et al. 2019; Li et al. 2018; Meissner et al. 2012; Wingen et al. 2018). In that way, the researchers could ascertain what steps to perform when witnessing a person with cardiac arrest after the training. Subsequently, the AHA or European Resuscitation Council questionnaire mainly was used to assess the adolescents’ CPR knowledge and skills pre- and post-training (Barsom et al. 2020; Nord et al. 2017; Pivač et al. 2020). The assessment included evaluation of scene safety and the consciousness of the victim, calling for emergency assistance, performing chest compressions and airway management. These outcomes influence the adolescents to increase their confidence around CPR, develop their competence and facilitate understanding of the diffusion of responsibility during CPR. Furthermore, the combination of the theory and didactics in training enables them to promptly address the needs of a cardiac arrest victim even though the scenarios they were assessed.

This systematic review revealed vast numbers of CPR school-based training interventions offered to adolescents, especially in Western countries. Tsai et al. (2019) followed up with the participants three months post-intervention and found that there was a slight drop of 1% of the participants’ CPR knowledge. That was the minimal duration of follow-up in all the included studies. Other follow-up periods of 6 months and 1 year post-intervention showed that overall, the participants’ CPR knowledge and CPR technical acquisition remained at a sufficient level. However, it is recommended to have refresher courses more frequently and in shorter intervals for optimal retention of the CPR skills and knowledge (Chien et al. 2020).

### Limitations of the study

The major limitation of this review was the lack of any study from the African continent that reported on school-based CPR training among the adolescents, which made it impossible to compare what training intervention strategy works best for resource-limited settings. The other limitation included not having much information that could realistically provide data of the adolescents providing CPR for a real-life scenario post-training.

### Implications and future research

Firstly, there should be an urgent and immediate need for CPR training among adolescents in Africa. Secondly, research should focus on the best implementation strategy that would be sustainable in a low-resource setting. Thirdly, high-quality RCTs would be needed to provide evidence-based intervention on the effectiveness of CPR training implemented within school settings to promote change in CPR knowledge, skills and behaviour among adolescents. Lastly, low- and middle-income countries should allocate resources and funds to purchase mannikins and other training materials that would help enhance CPR training among adolescents in schools.

## Conclusion

The findings from this study showed that CPR training within school settings effectively promoted a change in CPR knowledge and skills among adolescents. Hence, continuous training of CPR among schoolchildren should be encouraged by policymakers, school authorities, parents and teachers to optimise the prompt usage of the skills in any cardiac event.

## Figures and Tables

**FIGURE 1-A1:**
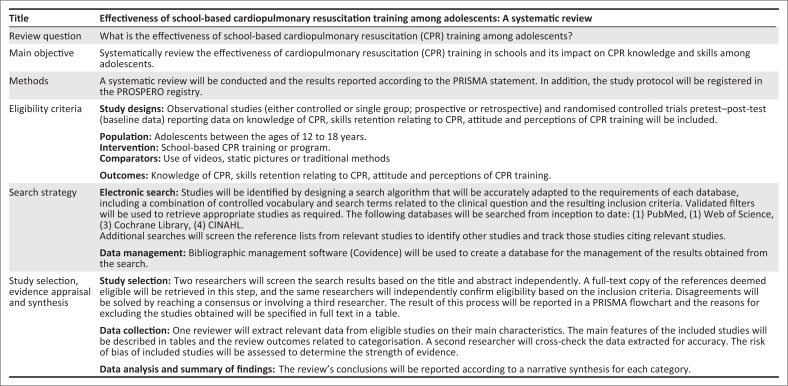
Summary of review protocol.
